# Structural Optimization of Carboxy-Terminal Phenylalanine-Modified Dendrimers for T-Cell Association and Model Drug Loading

**DOI:** 10.3390/pharmaceutics16060715

**Published:** 2024-05-27

**Authors:** Hiroya Shiba, Tomoka Hirose, Akinobu Sakai, Ikuhiko Nakase, Akikazu Matsumoto, Chie Kojima

**Affiliations:** 1 Department of Applied Chemistry, Graduate School of Engineering, Osaka Metropolitan University, 1-1 Gakuen-cho, Naka-ku, Sakai 599-8531, Osaka, Japan; 2 Department of Biological Science, Graduate School of Science, Osaka Metropolitan University, 1-1 Gakuen-cho, Naka-ku, Sakai 599-8531, Osaka, Japan

**Keywords:** dendrimer, drug delivery system, encapsulation, phenylalanine, T-cells

## Abstract

Dendrimers are potent nanocarriers in drug delivery systems because their structure can be precisely controlled. We previously reported that polyamidoamine (PAMAM) dendrimers that were modified with 1,2-cyclohexanedicarboxylic acid (CHex) and phenylalanine (Phe), PAMAM-CHex-Phe, exhibited an effective association with various immune cells, including T-cells. In this study, we synthesized various carboxy-terminal Phe-modified dendrimers with different linkers using phthalic acid and linear dicarboxylic acids to determine the association of these dendrimers with Jurkat cells, a T-cell model. PAMAM-*n*-hexyl-Phe demonstrated the highest association with Jurkat T-cells. In addition, dendri-graft polylysine (DGL) with CHex and Phe, DGL-CHex-Phe, was synthesized, and its association with Jurkat cells was investigated. The association of DGL-CHex-Phe with T-cells was higher than that of PAMAM-CHex-Phe. However, it was insoluble in water and thus it is unsuitable as a drug carrier. Model drugs, such as protoporphyrin IX and paclitaxel, were loaded onto these dendrimers, and the most model drug molecules could be loaded into PAMAM-CHex-Phe. PTX-loaded PAMAM-CHex-Phe exhibited cytotoxicity against Jurkat cells at a similar level to free PTX. These results suggest that PAMAM-CHex-Phe exhibited both efficient T-cell association and drug loading properties.

## 1. Introduction

The design of nanocarriers is important in drug delivery systems (DDSs). Precisely designed nanocarriers deliver bioactive compounds to target T-cells to enhance pharmacological effects and reduce side effects [1,2,3]. Various nanocarriers have been studied [4,5,6], and dendrimers are promising nano-sized materials. Because dendrimers are produced through stepwise reactions, their molecular weight, particle size, and surface charge can be controlled [7,8]. Dendrimers can also incorporate or modify various bioactive compounds within their internal space or at their surface [9,10]. Thus, dendrimers have been extensively studied as potential DDS nanocarriers. There are many kinds of dendrimers, such as polyamidoamine (PAMAM), polypropyleneimine, and polyester dendrimers [11,12,13]. Polylysine dendrimers and dendri-graft polylysines (DGLs) are also dendritic polymers, and because these are composed of lysine, they are useful as biomaterials with excellent biocompatibility [14,15]. PAMAM dendrimers and DGLs are commercially available, and PAMAM dendrimers have been extensively studied for anticancer drug and gene delivery [16,17].

T-cells play a vital role in our immune system, which determines the quantity and quality of the immune response against foreign invading substances or pathogens. Thus, the regulation of T-cell functions is important for the treatment and prevention of various diseases, which is attractive for immunotherapy [18,19,20,21]. For example, immune checkpoint inhibitors are used to enhance T-cell responses to diseased cells [22,23], and chimeric antigen receptor (CAR)-T-cells, which are genetically engineered T-cells, have been developed [24,25]. T-cell lymphocytic leukemia is one of the target diseased cells that should receive treatment. Thus, the establishment of a DDS for therapeutic materials to T-cells is required. Although antibodies and viruses have been studied for the delivery of therapeutic materials to T-cells, it remains challenging to efficiently deliver them inside the T-cells using nonviral materials [26].

**Figure 1:**
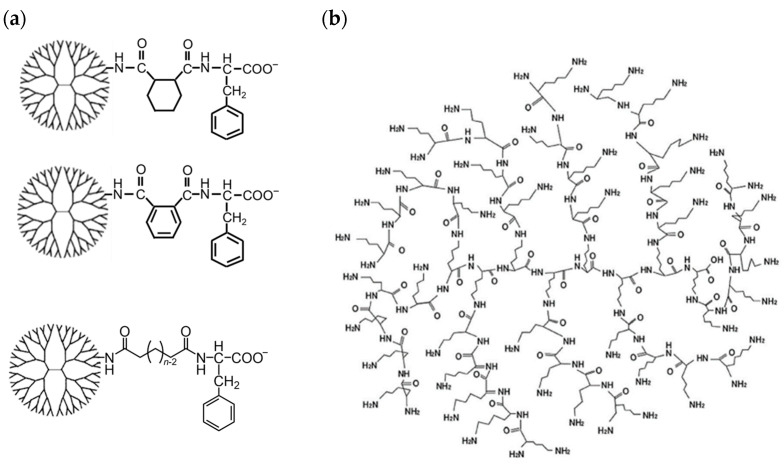
Structures of (**a**) carboxy-terminal phenylalanine (Phe)-modified PAMAM dendrimers used in this study and (**b**) DGL of G2.

Previously, we reported that PAMAM dendrimers with different anionic terminals containing carboxylate, sulfonate, or phosphate were highly accumulated in lymph nodes [27]. Although carboxy- and sulfo-terminal dendrimers did not efficiently associate with any immune cells, the dendrimers with both 1,2-cyclohexanedicarboxylic acid (CHex) and phenylalanine (Phe) at the termini (PAMAM-CHex-Phe and PAMAM-Phe-CHex) were highly associated with immune cells, including T-cells and their subsets [27,28]. We further revealed that these dendrimers were suitable for delivering protoporphyrin IX (PpIX) and plasmid DNA to T-cells [29,30]. It was previously reported that the Phe residues of the dendrimers influenced the association with T-cells, and it was determined that the highest T-cell association was at a 75% Phe density [29]. In this study, we attempted to optimize the dendrimer structure for the association with T-cells and model drug loading. Aromatic phthalic acid (Ph) and linear dicarboxylic acids with different lengths were used as the linker instead of CHex to evaluate the effect of the linker structure on the interaction with T-cells. We also synthesized DGLs modified with CHex and Phe to investigate the interaction with T-cells and elucidate the effect of the core structure (Figure 1). Furthermore, PpIX and paclitaxel (PTX) were used as model drugs, and the drug loading ability of these dendrimers was investigated.

## 2. Materials and Methods

### 2.1. Synthesis

PAMAM-CHex-Phe synthesized in our previous study was used [29]. PAMAM-Ph-Phe was synthesized, as shown in our previous report [31]. Briefly, 170 mg (12.0 µmol) of amino-functional ethylenediamine core PAMAM dendrimer of G4 (Sigma-Aldrich Co., St. Louis, MO, USA) was dissolved in dimethyl sulfoxide (DMSO)/*N*,*N*-dimethyl formamide (DMF) mixture (4 mL, 5/1 in vol ratio) and then excess phthalic anhydride (FUJIFILM Wako Pure Chemical Co., Osaka, Japan, 1.0 g, 6.8 mmol) and triethylamine (TEA, 600 μL, 4.3 mmol) were added to the solution. The mixture was stirred overnight at ambient temperature (approximately 25 °C), and the reaction mixture was dialyzed (MWCO 2k) in DMSO and then water for purification. PAMAM-Ph was obtained after lyophilization, whose yield was 198 mg (64%). Then, PAMAM-Ph (100 mg, 3.9 µmol) was dissolved in 5 mL of DMSO, and then 0.24 g (0.56 mmol) of L-Phe methyl ester hydrochloride (Phe-OMe·HCl, Nacalai Tesque, Inc., Kyoto, Japan), 0.20 g (0.53 mmol) of [benzotriazol-1-yloxy(dimethylamino)methylidene]-dimethylazanium hexafluorophosphate (HBTU, Watanabe Chemical Industries, Ltd., Hiroshima, Japan), and 70 µL (0.50 mmol) of TEA were added to the dendrimer solution and stirred for 4 days at ambient temperature. The dendrimer was dialyzed in DMSO and then methanol. PAMAM-Ph-Phe-OMe was obtained after lyophilization, whose yield was 123 mg (98%). Then, deprotection was performed, as follows. PAMAM-Ph-Phe-OMe (123 mg, 3.8 µmol) was dissolved in 4 mL of methanol, and 0.5 mL of 4 M NaOH methanol solution was added. After stirring for 3 h in an ice bath, the reaction mixture was dialyzed in water for purification. PAMAM-Ph-Phe was obtained after lyophilization, whose yield was 87 mg (71%).

PAMAM-C_6_-Phe was synthesized, as follows. An amount of 40 mg (2.8 µmol) of amino-terminal G4 PAMAM dendrimer was dissolved in DMSO, and about 140 equivalents of monomethyl suberate (Tokyo Chemical Industry Co., Ltd., Toyo, Japan), 100 equivalents of HBTU, and 100 equivalents of TEA were added to the dendrimer solution and stirred for 4 days at ambient temperature. The dendrimer solution was dialyzed in methanol for purification and lyophilized to obtain PAMAM-C_6_-OMe, whose yield was 71 mg (89%). Then, the deprotection of PAMAM-C_6_-OMe was performed, as described above. PAMAM-C_6_ was obtained after lyophilization, whose yield was 61 mg (92%). Then, PAMAM-C_6_ (55 mg, 2.1 µmol) was dissolved in 5 mL of DMSO and L-Phe benzyl ester 4-toluenesulfonate salt (Phe-OBzl·Tos, Peptide Institute, Inc., Osaka, Japan, 0.14 g, 0.35 mmol), HBTU (0.1 g, 0.27 mmol), and TEA (45 µL, 0.32 mmol) were added to the dendrimer solution and stirred for 4 days at ambient temperature. The dendrimer solution was dialyzed in methanol. Lyophilization was carried out to obtain PAMAM-C_6_-Phe-OBzl, whose yield was 78 mg (76%). Then, the deprotection of PAMAM-C_6_-Phe-OBzl was performed, as described above. PAMAM-C_6_-Phe was obtained after lyophilization (64.3 mg, 62%). PAMAM-C_4_-Phe and PAMAM-C_8_-Phe were synthesized in accordance with the procedure of PAMAM-C_6_-Phe by replacing monomethyl suberate with monomethyl adipate (Tokyo Chemical Industry) and monomethyl sebacate (Tokyo Chemical Industry), respectively. The yields of PAMAM-C_4_-Phe-OBzl, PAMAM-C_8_-Phe-OBzl, PAMAM-C_4_-Phe and PAMAM-C_8_-Phe were 66 mg (67%), 61 mg (57%), 59 mg (59%) and 49 mg (45%), respectively.

DGL-CHex-Phe was synthesized, as follows. An amount of 100 mg (11.6 µmol) of generation 2 (G2) of DGL (COLCOM, Montarnaud, France) was dissolved in 5 mL of 125 mM NaHCO_3_ aqueous solution, and then about 150 equivalents of cis-1,2-cyclohexanedicarboxylic anhydride (Tokyo Chemical Industry) were added. The pH of the DGL solution was adjusted to about 9 using 4 M NaOH aqueous solution. After stirring for 1 day at ambient temperature, the DGL solution was dialyzed in 125 mM NaHCO_3_ aqueous solution and then water. DGL-CHex was obtained after lyophilization (133.8 mg, 68%). Then, DGL-CHex (96.5 mg, 5.7 µmol) was dispersed in DMSO for 1 day and Phe-OBzl·Tos (176.4 mg, 0.41 mmol), HBTU (171.1 mg, 0.45 mmol), and TEA (113 µL, 0.82 mmol) were added to the DGL solution and stirred for 2 days at room temperature. DGL-CHex-Phe-OBzl was precipitated by adding HCl aqueous solution. After drying under vacuum conditions, DGL-CHex-Phe-OBzl was obtained, whose yield was 126 mg (81%). Then, deprotection of DGL-CHex-Phe-OBzl was performed, as described above. DGL-CHex-Phe was obtained after lyophilization (116 mg, 85%).

Fluorescein isothiocyanate (FITC, Tokyo Chemical Industry)-labeled PAMAM-CHex and PAMAM-CHex-Phe synthesized in our previous reports were used [29]. Other dendrimers were labeled with FITC in the same procedure as our previous report [29]. Briefly, 5–10 mg of dendrimers was dissolved in 0.5 mL of 100 mM NaHCO_3_ aqueous solution, and 6–10 equivalents of *N*-(2-aminoethyl) carbamic acid *tert*-butyl ester (Tokyo Chemical Industry) and 6 equivalents of 4-(4,6-dimethoxy-1,3,5-triazin-2-yl)-4-methylmorpholinium chloride (DMT-MM, FUJIFILM Wako Pure Chemical) were added to the dendrimer solutions and stirred overnight at ambient temperature. The dendrimer solutions were ultrafiltrated for purification by Amicon^®^Ultra (MWCO 3 kDa, Merck Millipore, Darmstadt, Germany) using 125 mM NaHCO_3_ aqueous solution and water. Then, the deprotection of *tert*-butoxycarbonyl (Boc) group by the treatment with 1 mL of trifluoroacetic acid (TFA, FUJIFILM Wako Pure Chemical) for 3 h in an ice bath was performed. After the removal of TFA and drying under vacuum conditions, these dendrimers were dissolved in 1 mL of DMSO. An amount of 15 equivalents of FITC to the dendrimer and excess TEA were added to the dendrimer solution. After stirring for two days at ambient temperature, the reaction mixtures were diluted with water until the concentration of DMSO was less than 2.5%. After purification by ultrafiltration (MWCO 3 kDa) and the following lyophilization, FITC-labeled dendrimers were obtained.

### 2.2. Characterization

Proton nuclear magnetic resonance (^1^H-NMR) spectra were recorded on a JNM-ECX (JEOL Ltd., Tokyo, Japan) spectrometer at a resonance frequency of 400 MHz at ambient temperature in glass tubes to estimate the bound numbers of Phe and the linker compound to the dendrimer. The UV–Vis spectra were measured by using a Jasco Model V630 UV/Vis spectrophotometer (JASCO Inc., Tokyo, Japan) to estimate the bound number of FITC to the dendrimer from the calibration curve and the absorbance at 513 nm in the spectra. The Log *p* (octanol/water partition coefficient) value was calculated by using ChemDraw (PerkinElmer Inc., Shelton, CT, USA).

### 2.3. Association of Dendrimers with Jurkat Cells

The association of dendrimers synthesized in this report with Jurkat cells was investigated by using GUAVA^®^ InCyte™ (Luminex, Austin, TX, USA), according to our previous report [29]. Briefly, each FITC-labeled PAMAM-CHex, PAMAM-CHex-Phe and PAMAM-Ph-Phe, PAMAM-C_4_-Phe, PAMAM-C_6_-Phe, PAMAM-C_8_-Phe, DGL-CHex, and DGL-CHex-Phe was added to RPMI (FITC 5 µM). Because DGL-CHex-Phe was not soluble in water, 500 µM of DGL-CHex-Phe-containing DMSO solution was prepared and diluted with RPMI. The dendrimer solutions were added to 1 × 10^5^ Jurkat cells and incubated for 3 h at 37 °C. Then, phosphate-buffered saline (PBS) was added to the cell suspension and centrifuged to collect the cells. After washing with PBS (400 µL) once, fluorescence-activated cell sorting (FACS) was performed to measure the mean green fluorescence intensity.

### 2.4. Adsorption of Dendrimers onto Liposomes

The adsorption of dendrimers onto liposomes was performed according to the method, as we previously reported [29]. Briefly, hydrogenated soy phosphatidylcholine (HSPC, NOF Corp., Tokyo, Japan, 10 mg/mL) dispersed in chloroform solution was dried overnight under vacuum conditions. Then, the dried solid was dissolved in 0.1 M 4-(2-hydroxyethyl)-1-piperazineethanesulfonic acid (HEPES) buffer (pH 7.4) and sonicated for 2 min using a bath sonicator (ASU-6, AS ONE Corp., Osaka, Japan) to obtain the dispersed liposomes (HSPC, 1.6 mg/mL). Then, 25 µL of the FITC-labeled PAMAM dendrimer-containing aqueous solutions (100 µM FITC) was mixed with 475 µL of the liposome solution and incubated for 3 h at 37 °C. The centrifugation (11,000 rpm for 15 min at 37 °C) was carried out to precipitate liposomes, and liposomes were washed with HEPES buffer. Then, chloroform/methanol (1/1, 0.5 mL) was added and shaken for 20 min at 37 °C to dissolve liposomes. The fluorescence intensity was measured to estimate the adsorption of these dendrimers to liposomes by using an FP-6200 spectrofluorometer (JASCO Inc.). The excitation and emission wavelengths were measured at 495 nm and 520 nm, respectively. The adsorption ratio of dendrimer to liposomes was calculated from the fluorescence intensity ratio of the solution before and after liposome treatment.

### 2.5. Model Structure of the Dendrimers

The molecular structure of the 4-terminal PAMAM dendrimers with Phe and linkers, such as CHex, Ph, and C_6_, in water was calculated using Spartan 08 (Wavefunction Inc., Irvine, CA, USA).

### 2.6. Encapsulation of Model Drugs in PAMAM Dendrimers

The loading of PpIX in dendrimers was performed according to the method described in our previous report [29]. Briefly, PpIX (Sigma-Aldrich) and each dendrimer were dissolved in DMF, and mixed to adjust the mole ratio of PpIX/dendrimer at 10/1. The solution was then evaporated, dried under vacuum conditions, and dissolved in water (dendrimer 0.1 mM). The loaded PpIX in the dendrimer was collected from the supernatant after centrifugation, because free PpIX was insoluble in water. The UV–Vis spectrum of each supernatant was measured to estimate the amount of encapsulated PpIX. The amount of loaded PpIX was calculated from the calibration curve and absorbance at 406 nm.

The loading of PTX in the dendrimers was performed according to the method described in our previous report [32]. Briefly, PTX (Tokyo Chemical Industry) and each dendrimer were dissolved in methanol at a 5:1 mol ratio of PTX:dendrimer. The solution was then evaporated, dried under vacuum conditions, and dissolved in water (dendrimer 0.1 mM). PTX loaded in the dendrimer was collected from the supernatant after centrifugation. The amount of encapsulated PTX was estimated by HPLC analysis using the calibration curve. The same experiment was carried out by replacing water used after drying with saline as a solvent. After the incubation at room temperature (approximately at 25 °C) for 24 h, the supernatant was collected to measure the retained PTX in the solution.

### 2.7. Cytotoxicity of the PTX-Loaded Dendrimer

The cytotoxicity of the PTX-loaded PAMAM-CHex-Phe was examined, according to our previous report [30]. Briefly, free PTX and the PTX-loaded PAMAM-CHex-Phe prepared in Section 2.6 were added to 1 × 10^4^ Jurkat cells in 100 µL of RPMI medium at a PTX concentration of 10 nM. After incubation for 48 h, cells were washed with PBS. Then, 5 mg/mL of 3-(4,5-dimethylthiazol-2-yl)-2,5-diphenyl-2H-tetrazolium bromide (MTT)-containing PBS solution (10 µL) and RPMI (90 µL) was added to the cells and incubated for 3 h. After centrifugation at 3000 rpm for 5 min, the supernatant was removed, and 0.1 M HCl-containing isopropyl alcohol (200 µL) was added to dissolve cells. The absorbance at 570 nm was measured to estimate the amount of cell viability. The cell viability (%) was calculated from the percentage of the absorbance of cells treated with the sample to that of intact cells. The cytotoxicity of Jurkat cells treated with 10 µM of the dendrimer without PTX for 24 h was also examined.

## 3. Results and Discussion

### 3.1. Synthesis of Carboxy-Terminal Phe-Modified Dendrimers

**Figure 2:**
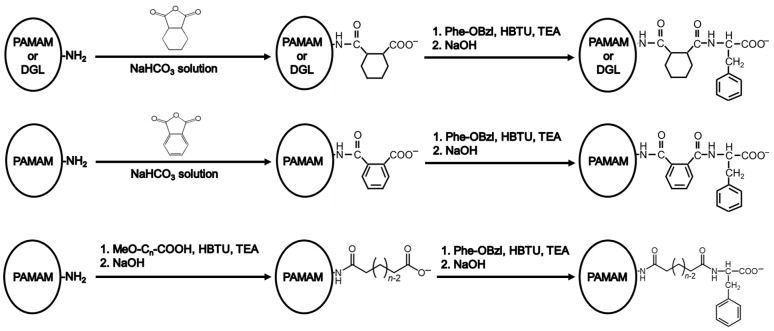
Synthetic scheme of carboxy-terminal phenylalanine (Phe)-modified PAMAM dendrimers and DGL with CHex (**top**), Ph (**middle**), and C_n_ (**bottom**) linkers. MeO-C_n_-COOH are monomethyl adipate (*n* = 4), monomethyl suberate (*n* = 6), and monomethyl sebacate (*n* = 8), respectively.

Firstly, carboxy-terminal Phe-modified PAMAM dendrimers with different linkers, such as CHex, Ph, C_4_, C_6_, and C_8_, and DGL-CHex-Phe were synthesized (Figure 2). The amino-terminal PAMAM G4 dendrimer was reacted with an excess of acid anhydrides (CHex and Ph) or linear dicarboxylic acid monoesters (C_4_, C_6_, and C_8_) to conjugate the different linkers. The hydrolysis of the ester group was carried out for dendrimers reacted with linear dicarboxylic acid monoesters. Then, Phe with a protective group at the carboxy-terminal was conjugated to the carboxy-termini of the dendrimers. The subsequent deprotection of the ester group was performed. DGL-CHex-Phe was synthesized using the same procedure as for PAMAM-CHex-Phe by replacing PAMAM with DGL. For comparison, carboxyl-terminal dendrimers without Phe, PAMAM-CHex, and DGL-CHex were also synthesized. The synthesized dendrimers were characterized by ^1^H NMR spectroscopy. The average bound numbers of CHex, Ph, C_4_, C_6_, and C_8_ were evaluated from the integral ratios of the signals at around 1.1–1.9 ppm for CHex, 7.2–7.8 ppm for Ph, 1.5–2.2 ppm for C_4_–C_8_, 4.2 ppm for Phe, 2.2 ppm for PAMAM dendrimer, and 4.1 ppm for DGL (Figures S1–S4), which are listed in Table 1. The terminal numbers of PAMAM G4 and DGL G2 were 64 and 48, respectively. The linker compounds and Phe were conjugated to the dendrimer at all termini. The dendrimers were then labeled with a green fluorescent dye, FITC. Two to nine FITC molecules were conjugated to each dendrimer after introducing small amounts of amino groups to the dendrimer according to the method described in our previous report (Table 1) [29]. These FITC-labeled dendrimers were used to investigate the associations with the cells and liposomes. The molecular weights of these dendrimers are also listed in Table 1. The molecular weights of the dendrimers increased after the Phe modification. The Phe-modified PAMAM dendrimers were similar, regardless of the linker compounds. The DGLs were smaller than the PAMAM dendrimers.

### 3.2. Association of Carboxy-Terminal Phe-Modified Dendrimers with Jurkat Cells

**Figure 3:**
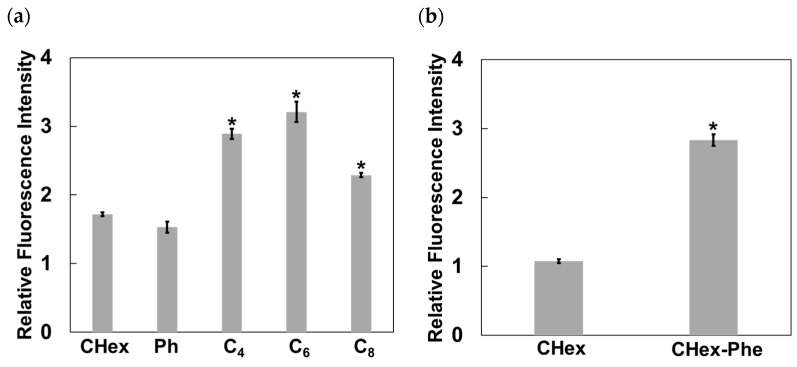
Association of dendrimers with Jurkat cells. (**a**) Fluorescence intensity of PAMAM-R-Phe normalized to PAMAM-CHex. R means CHex, Ph and C_n_ linkers. (**b**) Fluorescence intensity of DGL-CHex and DGL-CHex-Phe normalized to PAMAM-CHex. * *p* < 0.05 vs. PAMAM-CHex.

**Figure 4:**
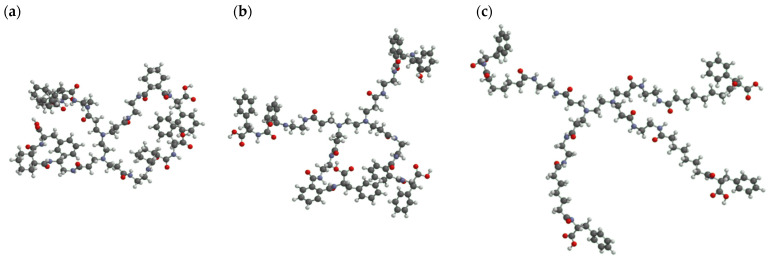
Structure of carboxy-terminal phenylalanine (Phe)-modified PAMAM dendrimers via different linkers with four termini. (**a**) PAMAM-CHex-Phe, (**b**) PAMAM-Ph-Phe, and (**c**) PAMAM-C_6_-Phe. White, black, blue, and red balls correspond to hydrogen, carbon, nitrogen, and oxygen atoms, respectively.

**Figure 5:**
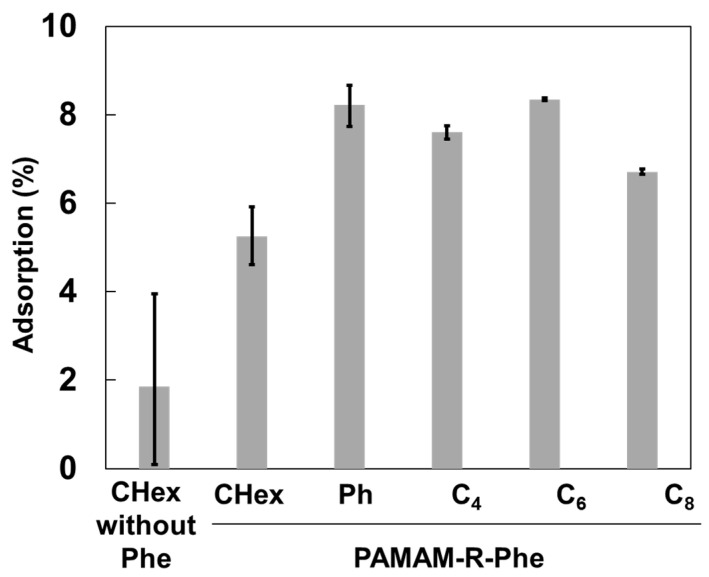
Adsorption of carboxy-terminal phenylalanine (Phe)-modified dendrimers to liposomes via different linkers after the 3 h-incubation.

The association of dendrimers with Jurkat cells, as a T-cell model, was examined. The mean fluorescence intensity of the Jurkat cells treated with FITC-labeled PAMAM dendrimers was measured by FACS. Figure 3a shows that the mean fluorescence intensity of the carboxy-terminal Phe-modified PAMAM dendrimers containing linear C_4_, C_6_, and C_8_ was higher than that of PAMAM-CHex-Phe and PAMAM-Ph-Phe. This indicates that the linear dicarboxylic acid-containing dendrimers associate with Jurkat cells more efficiently than PAMAM-CHex-Phe and PAMAM-Ph-Phe. PAMAM-C_6_-Phe exhibited the highest fluorescence intensity. Our previous results indicate that PAMAM-CHex-Phe was internalized into Jurkat cells, which were observed by confocal microscopy [28]. Thus, it is possible that these dendrimers were also internalized into Jurkat cells. Linear linkers were effective in inducing an association with T-cells, although our previous reports indicate that PAMAM-C_2_-Phe (PAMAM-Suc-Phe) was not associated with Jurkat cells [28]. Therefore, T-cell association can be optimized by adjusting the length of the alkyl chain at the dendrimer termini. The linkers of PAMAM-CHex-Phe, PAMAM-Ph-Phe, and PAMAM-C_6_-Phe have the same number of carbons. The Log *p* values of PAMAM-CHex-Phe, PAMAM-Ph-Phe, and PAMAM-C_6_-Phe, whose dendrimer contains four termini, were calculated as a model and were estimated as 6.8, −0.8, and 4.1, respectively. This indicates that the hydrophobicity of PAMAM-CHex-Phe and PAMAM-C_6_-Phe was similar, which was more hydrophobic than PAMAM-Ph-Phe. The association of PAMAM-Ph-Phe and PAMAM-CHex-Phe with Jurkat cells was similar despite the difference in Log *p* values. PAMAM-C_6_-Phe exhibited a greater association with Jurkat cells than PAMAM-CHex-Phe despite having similar Log *p* values. These indicate that the Log *p* values are not the main factor involved in the association with T-cells. Ishii et al. reported that the modification of nanoparticle surfaces with polyethylene glycol (PEGs) of different lengths enhanced ligand mobility and target recognition [33]. In our previous report, we demonstrated that the Phe density at the dendrimer terminus affects the cellular interactions in PAMAM-CHex-Phe [29]. The most stable molecular structures of PAMAM-CHex-Phe, PAMAM-Ph-Phe, and PAMAM-C_6_-Phe, whose dendrimer contains four termini, were calculated and presented in Figure 4. PAMAM-C_6_-Phe has a more spread structure than the others, which suggests that the Phe residues of PAMAM-C_6_-Phe readily interact with Jurkat cells. The interaction of these dendrimers with the cellular membranes was investigated using liposomes as a model (Figure 5). Although adsorption to the liposomes was slightly increased by adding Phe and changing the linkers, it was less than 10%. Thus, it is likely that these dendrimers did not directly interact with the lipid membranes. Further investigation is required to elucidate the cell association mechanism of these dendrimers.

Figure 3b shows the mean fluorescence intensity of DGL-CHex and DGL-CHex-Phe. The fluorescence intensity of DGL-CHex without Phe was similar to that of PAMAM-CHex without Phe. The fluorescence intensity of DGL-CHex-Phe was higher than that of PAMAM-CHex-Phe. Although DGL-CHex-Phe exhibited excellent cell association properties, it was not soluble in water. Thus, DGL-CHex-Phe was unsuitable as a drug carrier.

### 3.3. Model Drug Loading Using Carboxy-Terminal Phe-Modified Dendrimers with Various Linkers

**Figure 6:**
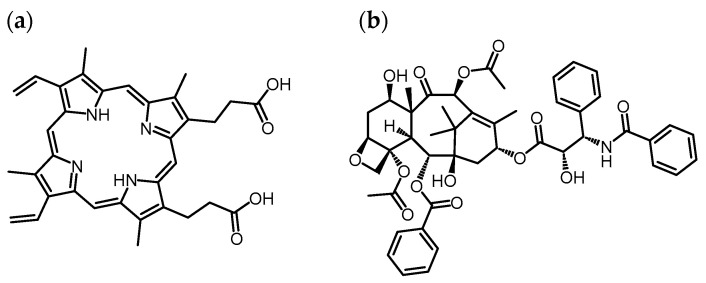
Chemical structure of (**a**) PpIX and (**b**) PTX.

**Figure 7:**
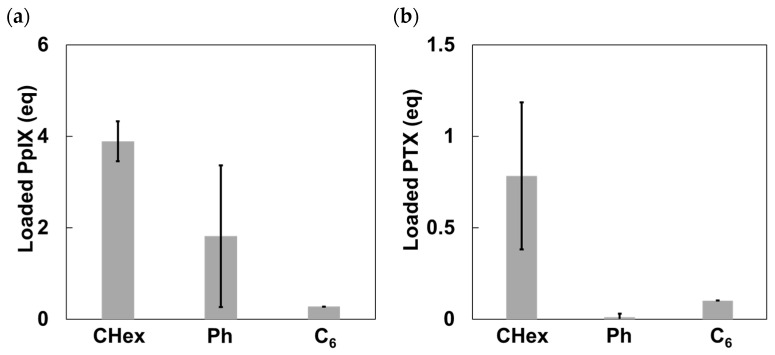
Loading of (**a**) PpIX and (**b**) PTX into PAMAM-CHex-Phe, PAMAM-Ph-Phe, and PAMAM-C_6_-Phe in water.

The drug loading ability of nanocarriers as a DDS is important. PpIX and PTX were used as model drugs in this study (Figure 6). PpIX is a water-insoluble photosensitizer that is beneficial for photodynamic therapy [29]. PTX is a water-insoluble anticancer agent [32]. It was recently reported that PTX exhibited some immune-modulating effects [34]. Thus, the delivery of these drugs into T-cells is useful for the treatment of T-cell leukemia as well as immunotherapy. The drug loading ability of PAMAM-CHex-Phe, PAMAM-Ph-Phe, and PAMAM-C_6_-Phe was examined in water, as previously reported [29,32]. When 10 equivalents of PpIX were added to each dendrimer, 3.9, 1.8, and 0.3 molecules of PpIX were loaded into PAMAM-CHex-Phe, PAMAM-Ph-Phe, and PAMAM-C_6_-Phe, respectively (Figure 7a). When five equivalents of PTX were added to each dendrimer, 0.78, 0, and 0.10 molecules of PTX were loaded into PAMAM-CHex-Phe, PAMAM-Ph-Phe, and PAMAM-C_6_-Phe, respectively (Figure 7b). PAMAM-CHex-Phe could encapsulate more PpIX and PTX than the other dendrimers, which suggests that the linker also affected the drug loading ability. Although PAMAM-C_6_-Phe was efficiently associated with cells, the drug loading ability was low. In our previous report, we revealed that the encapsulation of guest molecules into PAMAM dendrimers was based on electrostatic and hydrophobic interactions [29]. Because PAMAM-Ph-Phe is more hydrophilic than PAMAM-CHex-Phe and PAMAM-C_6_-Phe, as estimated from their Log *p* values, the interaction of PAMAM-Ph-Phe with the hydrophobic drug molecules was possibly suppressed. It is likely that the spread-out structure of PAMAM-C_6_-Phe, as shown in Figure 4, suppressed the interaction with drug molecules. More PpIX was encapsulated in these dendrimers than PTX because PpIX has a carboxy group that can interact with the inner tertiary amines of these dendrimers. The water solubility of these molecules was greatly enhanced when bound to the dendrimers. In particular, the water solubility of PTX was extremely low (reported as 0.35 μM) [35]. However, the water solubility of PTX was 78 μM in the aqueous solution of PAMAM-CHex-Phe, which is an increase of about 200 times. Thus, PAMAM-CHex-Phe not only enhanced the water solubility of hydrophobic molecules but was also associated with T-cells, which are the most useful properties for delivering small drug molecules to T cells.

### 3.4. Drug Action of PTX-Loaded PAMAM-CHex-Phe

We examined the drug action of the PTX-loaded PAMAM-CHex-Phe, the most promising drug–dendrimer complex, to Jurkat cells by the MTT cytotoxicity assay. The cytotoxicity of free PTX and PAMAM-CHex-Phe without PTX against Jurkat cells was also examined. Table 2 shows that the cell viability of Jurkat cells treated with PTX-loaded PAMAM-CHex-Phe was 28%, which was similar to that of free PTX. The cell viability of 10 μM PAMAM-CHex-Phe without PTX was 90%, although the dendrimer concentration was much higher than the PTX-loaded PAMAM-CHex-Phe. This indicates that PAMAM-CHex-Phe itself is essentially not cytotoxic. These suggest that PTX loaded in PAMAM-CHex-Phe worked as a similar drug action to free PTX. The retention of PTX in the dendrimer was also investigated. Before and after 24 h incubation in saline, the PTX concentration in the supernatant was measured. The PTX loaded in the dendrimer in saline was almost the same in water. Only 20% of PTX was retained in the supernatant after 24 h, but the concentration of PAMAM-CHex-Phe in the supernatant was almost unchanged. These suggest that the drug was released from the dendrimer. Our results suggest that PAMAM-CHex-Phe has potential as a small drug nanocarrier for direct delivery to T-cells.

## 4. Conclusions

We synthesized and characterized carboxy-terminal Phe-modified dendrimers with different linkers and cores to investigate the effect of the dendrimer structure to the association with Jurkat cells and model drug loading. PAMAM dendrimers with linear linkers exhibited a higher association with T-cells than dendrimers with cyclic linkers. DGL-CHex-Phe demonstrated a higher association with T-cells than PAMAM-CHex-Phe. This indicates that the linker and core structure of the dendrimer are factors involved in the association with T-cells. In addition, the dendrimer structure also affected the drug loading ability. PAMAM-CHex-Phe exhibited the greatest drug loading ability for PpIX and PTX, whose water solubility was drastically improved. PTX-loaded PAMAM-CHex-Phe exhibited similar cytotoxicity against Jurkat cells to free PTX, but PAMAM-CHex-Phe itself did not exhibit any significant cytotoxicity. These results suggest that PAMAM-CHex-Phe showed efficient cell association and drug loading properties, thus making it a potent small molecular drug carrier for direct drug delivery to T-cells. Recently, PTX has shown immune-modulating effects as well as anticancer effects [34]. PTX-loaded PAMAM-CHex-Phe may be used to stimulate immune cells as well as for the treatment of T-cell leukemia. Our previous papers indicated that carboxy-terminal Phe-modified dendrimers were associated with various kinds of immune cells [27,28,29]. The targeting property is required for the applications to DDS, which remains to be investigated.

## Figures and Tables

**Table 1:** Carboxy-terminal Phe-modified dendrimers used in this study.

**Dendrimer ^1^**	**Bound Numbers**			Molecular Weight (kDa) ^3^
	**Phe**	**Linker**	**FITC**	
PAMAM-CHex ^2^	0	64 (CHex)	4.0	24.0
PAMAM-CHex-Phe ^2^	64	64 (CHex)	7.0	33.5
PAMAM-Ph-Phe	59	64 (Ph)	3.7	32.4
PAMAM-C_4_-Phe	64	64 (C_4_)	2.0	31.7
PAMAM-C_6_-Phe	64	64 (C_6_)	8.0	33.5
PAMAM-C_8_-Phe	64	64 (C_8_)	7.0	35.3
DGL-CHex	0	48 (CHex)	9.3	17.0
DGL-CHex-Phe	48	48 (CHex)	4.5	24.1

^1^ The numbers of terminal groups of PAMAM and DGL are 64 and 48, respectively. ^2^ Refer to our previous reports [29]. ^3^ Calculated molecular weight of each dendrimer without FITC.

**Table 2:** Cell viability of free PTX, PTX-loaded PAMAM-CHex-Phe, and PAMAM-CHex-Phe.

Compound	Concentration		Incubation Time	Cell Viability (%)
	PTX	Dendrimer		
free PTX	10 nM	-	48 h	23 ± 14%
PTX-loaded PAMAM-CHex-Phe	10 nM	13 nM	48 h	28 ± 2%
PAMAM-CHex-Phe	-	10,000 nM	24 h	91 ± 20%

## Data Availability

Data supporting the findings of this study are available from the corresponding author upon reasonable request.

## Supplementary Materials

The following supporting information can be downloaded at: https://www.mdpi.com/article/10.3390/pharmaceutics16060715/s1, Figure S1: ^1^H NMR spectra of (a) PAMAM-C_8_-Phe, (b) PAMAM-C_6_-Phe, (c) PAMAM-C_4_-Phe in D_2_O containing NaOD; Figure S2: ^1^H NMR spectrum of PAMAM-CHex-Phe in D_2_O containing NaOD; Figure S3: ^1^H NMR spectrum of PAMAM-Ph-Phe in D_2_O containing NaOD; Figure S4: ^1^H NMR spectra of (a) DGL-CHex-Phe and (b) DGL-CHex in D_2_O containing NaOD.
