# Supplementary material for: Structural Optimization of Carboxy-Terminal Phenylalanine-Modified Dendrimers for T-Cell Association and Model Drug Loading
Source: Pharmaceutics. 2024 May 27;16(6):715. doi: 10.3390/pharmaceutics16060715 (PMC11206903; doi:10.3390/pharmaceutics16060715)
Supplement: Supplementary file 1 [file pharmaceutics-16-00715-s001.zip › pharmaceutics-3013365-supplementary.pdf]

Supplementary Information

Structural Optimization of Carboxy-Terminal Phenylalanine-Modified Dendrimers for T-Cell Association and Model Drug Loading

Hiroya Shiba, Tomoka Hirose, Akinobu Sakai, Ikuhiko Nakase, Akikazu Matsumoto and Chie Kojima

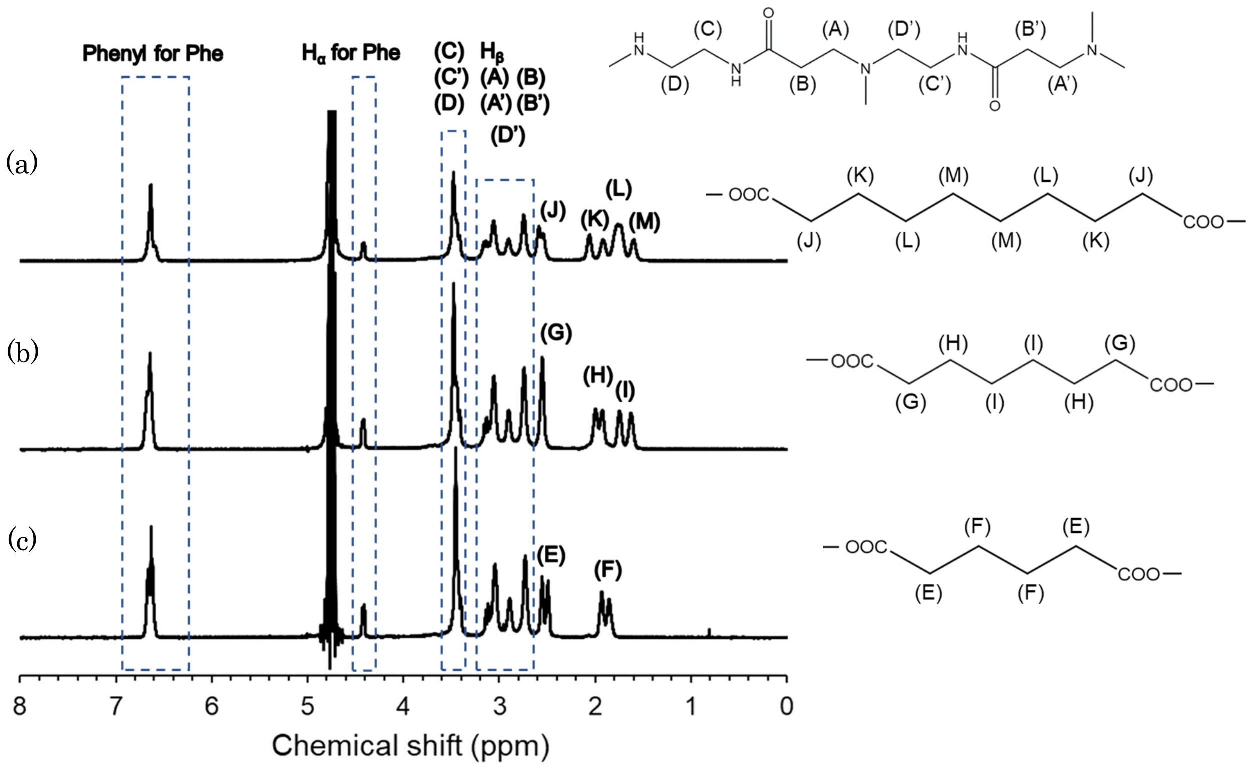

Figure S1. ^{1}H NMR spectra of (a) PAMAM-C_8-Phe, (b) PAMAM-C_6-Phe, (c) PAMAM-C_4-Phe in D_2O containing NaOD.

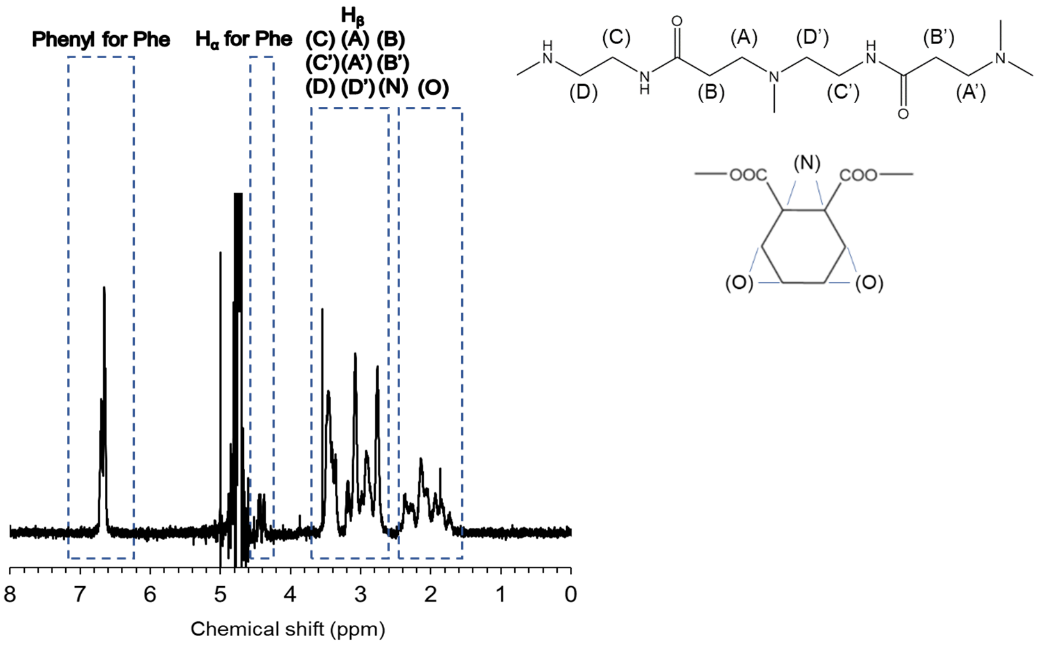

Figure S2. ^{1}H NMR spectrum of PAMAM-CHex-Phe in D_2O containing NaOD.

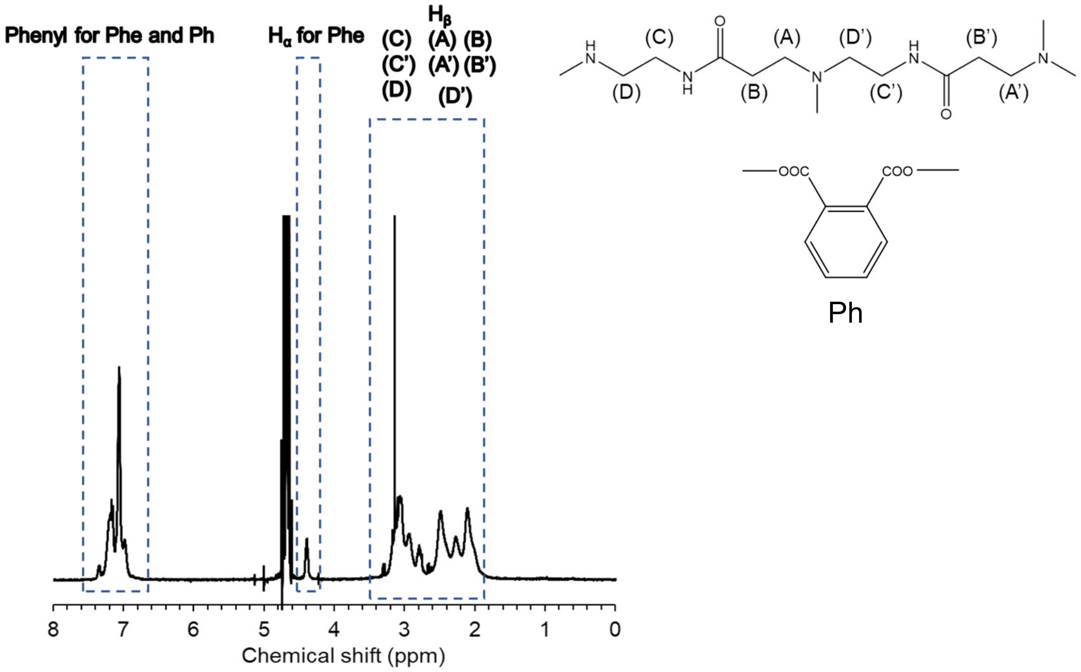

Figure S3. ^{1}H NMR spectrum of PAMAM-Ph-Phe in D_2O containing NaOD.

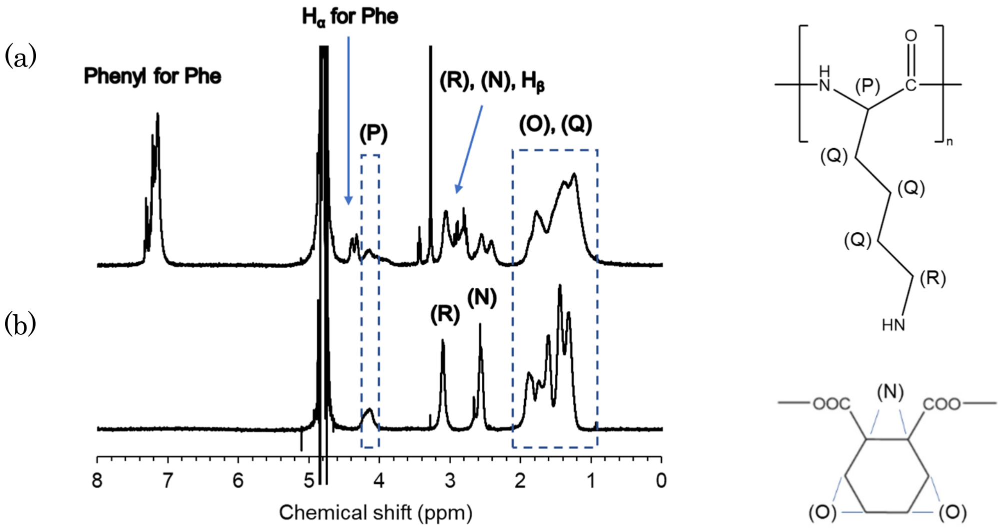

Figure S4. ^{1}H NMR spectra of (a) DGL-CHex-Phe and (b) DGL-CHex in D_2O containing NaOD.
